# Investigating the Host Binding Signature on the *Plasmodium falciparum* PfEMP1 Protein Family

**DOI:** 10.1371/journal.ppat.1002032

**Published:** 2011-05-05

**Authors:** Joel H. Janes, Christopher P. Wang, Emily Levin-Edens, Inès Vigan-Womas, Micheline Guillotte, Martin Melcher, Odile Mercereau-Puijalon, Joseph D. Smith

**Affiliations:** 1 Department of Global Health, University of Washington, Seattle, Washington, United States of America; 2 Seattle Biomedical Research Institute, Seattle, Washington, United States of America; 3 Institut Pasteur, Unité d'Immunologie Moléculaire des Parasites, Paris, France; 4 CNRS URA 2581, Paris, France; Weill Medical College of Cornell University, United States of America

## Abstract

The *Plasmodium falciparum* erythrocyte membrane protein 1 (PfEMP1) family plays a central role in antigenic variation and cytoadhesion of *P. falciparum* infected erythrocytes. PfEMP1 proteins/*var* genes are classified into three main subfamilies (UpsA, UpsB, and UpsC) that are hypothesized to have different roles in binding and disease. To investigate whether these subfamilies have diverged in binding specificity and test if binding could be predicted by adhesion domain classification, we generated a panel of 19 parasite lines that primarily expressed a single dominant *var* transcript and assayed binding against 12 known host receptors. By limited dilution cloning, only UpsB and UpsC *var* genes were isolated, indicating that UpsA *var* gene expression is rare under *in vitro* culture conditions. Consequently, three UpsA variants were obtained by rosette purification and selection with specific monoclonal antibodies to create a more representative panel. Binding assays showed that CD36 was the most common adhesion partner of the parasite panel, followed by ICAM-1 and TSP-1, and that CD36 and ICAM-1 binding variants were highly predicted by adhesion domain sequence classification. Binding to other host receptors, including CSA, VCAM-1, HABP1, CD31/PECAM, E-selectin, Endoglin, CHO receptor “X”, and Fractalkine, was rare or absent. Our findings identify a category of larger PfEMP1 proteins that are under dual selection for ICAM-1 and CD36 binding. They also support that the UpsA group, in contrast to UpsB and UpsC *var* genes, has diverged from binding to the major microvasculature receptor CD36 and likely uses other mechanisms to sequester in the microvasculature. These results demonstrate that CD36 and ICAM-1 have left strong signatures of selection on the PfEMP1 family that can be detected by adhesion domain sequence classification and have implications for how this family of proteins is specializing to exploit hosts with varying levels of anti-malaria immunity.

## Author Summary

The malaria parasite *Plasmodium falciparum* persists in the human host partly by avoiding elimination in the spleen during blood stage infection. This strategy depends principally upon members of the large and diverse PfEMP1 family of proteins that are exported to the surface of infected erythrocytes. PfEMP1 proteins are important targets for host protective antibody responses and encode binding to several different host receptor proteins. Switches in PfEMP1 expression allow parasites to evade host antibodies and may precipitate severe disease when infected erythrocytes accumulate in brain or placenta. Consequently, the severity of malaria infection may depend on the type of PfEMP1 protein expressed. In this study, we employ a representative panel of distinct PfEMP1 types and host receptor proteins to demonstrate that CD36 and ICAM-1 binding properties of full-length PfEMP1 are highly predicted by their domain composition. We also find that CD36 binding is under strong selection in many PfEMP1 proteins, but that a group of PfEMP1s associated with more severe infections does not bind CD36 and may utilize alternative means to sequester infected erythrocytes. These findings have implications for understanding the molecular basis for severe malaria.

## Introduction

*Plasmodium falciparum* erythrocyte membrane protein 1 (PfEMP1) is a clonally variant adhesion protein that mediates binding of infected erythrocytes (IE) to blood microvasculature and other host cells [1]. Adherence of IEs to microvascular endothelium is a major virulence factor and, in conjunction with the related phenomenon of rosetting with uninfected erythrocytes, prevents parasitized erythrocyte circulation to the spleen where parasites may be destroyed [2]. Each parasite strain encodes ∼60 PfEMP1 proteins, or *var* genes, which are expressed in a mutually exclusive fashion [3], [4]. Switches in *var* gene expression enable infected erythrocytes to evade host immunity and may modify disease manifestations by changing parasite binding tropism [5]–[7].

Efforts to unravel the role of PfEMP1 proteins in disease are complicated by the vast diversity of *var* genes. Each parasite has a diverse repertoire of genes, and there is limited overlap of repertoires between parasite genomes [8]–[10]. However, genes can be classified into three main subfamilies denoted Groups A, B, and C [11], plus three unusual strain-transcendent variants (*var1csa*, *var2csa*, and type 3 *var*) [12]–[15]. The *var* gene subfamilies possess distinctive upstream flanking regions termed UpsA, UpsB, and UpsC and are found in characteristic locations in the subtelomeric or central regions of chromosomes [4], [9], [11], [12]. It has been hypothesized that *var* gene organization may contribute to a gene recombination hierarchy that influences gene function and evolution [1].

A number of studies have sought to correlate specific parasite adhesion traits with disease outcome [16]–[19]. To date, at least 12 host receptors have been reported to mediate *P. falciparum* IE binding [20]. CD36 binding is the most common adhesion trait in the parasite population, followed by intercellular adhesion molecule 1 (ICAM-1) [17], [19]. These two receptors can synergize under flow conditions to mediate infected erythrocyte binding to microvasculature endothelium [21]–[23]. Most other binding properties appear to be rarer or have not been studied in more than one or a few parasite isolates. ICAM-1 binding has been associated with cerebral malaria in some studies [17], [24], but not in others [19], [25]. In addition, infected erythrocyte rosetting, or binding of parasitized red blood cells to uninfected red blood cells, has been associated with disease severity in African children [26]–[28]. The clearest disease association is placental malaria, in which parasites express the unusually strain-transcendent VAR2CSA PfEMP1 protein and adhere to chondroitin sulfate A (CSA) in the placenta [14], [29]. VAR2CSA is a leading candidate for a pregnancy malaria vaccine and a paradigm for syndrome-specific anti-disease vaccine efforts.

Although the molecular basis for other adhesion-based complications of *P. falciparum* is less established than for pregnancy malaria, several observations suggest the antigenic diversity of severe malaria isolates may also be limited. For instance, immunity to severe malaria appears to be acquired after relatively few infections [30], [31]. In addition, isolates from severe malaria cases appear to express a relatively restricted variant antigen surface repertoire [32]–[34]. Furthermore, seroepidemiological and *var* transcriptional profiling studies suggest that UpsA variants are more commonly expressed in young African children with limited immunity and in severe malaria infections [35]–[39]. Therefore it is possible the UpsA group has become specialized to exploit individuals with limited anti-malaria immunity, and it is important to understand what may account for this expression profile.

To gain insight into PfEMP1 binding properties, sequence classification has been performed [40]. The extracellular binding region of PfEMP1 proteins is comprised of 2–7 receptor-like domains called Duffy Binding-Like (DBL) and Cysteine Rich Interdomain Region (CIDR) [41], [42]. DBL and CIDR domains are classified into different major types (α to ε) and sub-types by sequence criteria [10], [40]. PfEMP1 proteins can be further subdivided by protein architecture into small proteins with a four-domain extracellular binding region (DBL-CIDR-DBL-CIDR) and large proteins with a more complex domain composition [43]. By comparison to other groups, nearly all of the UpsA proteins are in the large protein category [9], [10]. The best characterized binding interactions are between CIDR::CD36 and DBLβ::ICAM-1 [44]–[46]. In a repertoire-wide binding comparison with CIDR recombinant proteins, the majority of proteins encoded CD36 binding function, except for the UpsA group, which had different CIDR sequence types than the UpsB and UpsC groups [11], [12], [46]. UpsA proteins may also be under less selection to bind ICAM-1, as 7 of 23 DBLβ domains from the IT4 parasite strain bound ICAM-1, but none of the 9 DBLβ domains tested from the UpsA group were ICAM-1 binders [44]. However, using a different binding analysis in a BioPlex system, only a single DBLβ recombinant protein from the 3D7 parasite strain bound ICAM-1, and it was from an UpsA protein [45]. UpsA proteins have also been reported to bind ICAM-1 (PFD1235w) and PECAM-1 (PF11_0008) [47]. Taken together, sequence and binding analysis suggest the UpsA group forms a preferential gene recombination group that is under less selection to bind the primary microvasculature receptor CD36. Furthermore, it is possible UpsA genes may have evolved specialized binding properties that contribute to their preferential expression in the malaria non-immune.

While sequence and binding analysis of isolated domains have provided significant insights into PfEMP1 function, few binding predictions have been confirmed for native proteins at the IE surface, and it is not yet established whether binding differences truly exist between *var* gene subfamilies. Furthermore, it is possible that recombinant protein binding properties may be modified by adjacent domains [48] or may not extrapolate to the native PfEMP1 molecule [49]. Thus, there remain significant uncertainties in our ability to predict IE binding, and there is still limited understanding of how host selection is shaping the PfEMP1 variant antigen repertoire for parasite survival and transmission. For this study, we generated a large panel of cloned parasite lines from the cytoadhesive IT4/FCR3 parasite strain and selected three highly enriched UpsA parasite lines with specific monoclonal antibodies. This panel was employed to both investigate the major host selection binding pressures operating on the protein family and to evaluate binding predictions based on sequence information and isolated domain binding assays.

## Results

### Generation of a panel of cloned parasite lines from a cytoadhesive parasite line

**Figure 1:**
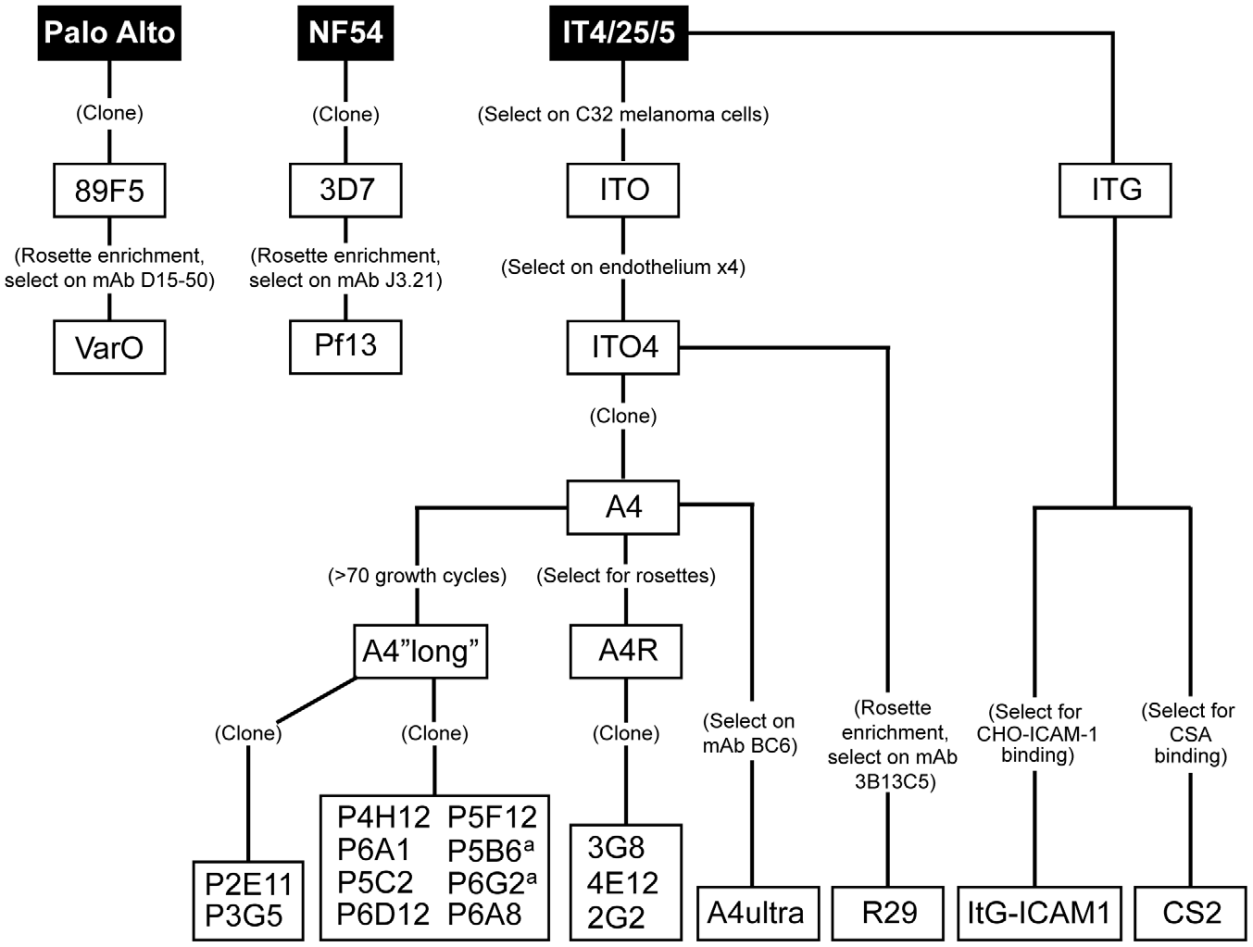
Derivation of parasite lines for phenotypic analysis. A panel of 19 *P. falciparum* parasite lines (clear boxes) was generated from parental laboratory lines (black boxes) using limited dilution cloning or various selection techniques. All parasite lines express a unique predominant *var* transcript by qRT-PCR or monoclonal antibody reactivity except for two lines (a) that both express *IT4var31.*

**Figure 2:**
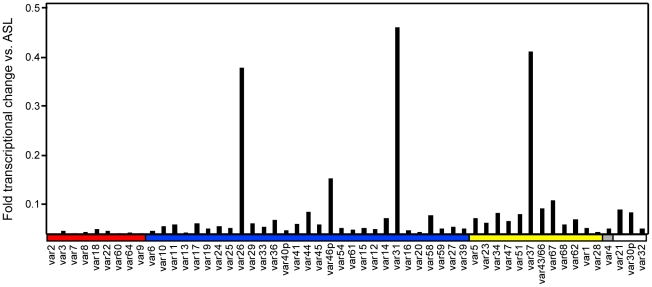
Analysis of *var* gene transcription in the long-term A4 parasite clone culture. A4 parasites were grown under continuous culture in the absence of selection for more than 70 parasite divisions, and RNA was harvested. The profile of *var* gene expression was measured by qRT-PCR, using specific primers to the IT4*var* genes. After 70 cycles, the parasite culture had nearly completely switched away from *IT4var14/A4var* and predominantly expressed three *var* transcripts (*IT4var26*, *IT4var31*, and *IT4var37*), plus many genes at lower levels. Gene expression was normalized to the housekeeping control gene adenylosuccinate lyase (*asl*). The A4 primer set is arranged along the *x*-axis by Ups category: UpsA (red), UpsB (blue), UpsC, (yellow), and UpsE (gray) groups, as well as three genes for which the Ups type has not been determined (white).

To create a panel of parasites for phenotypic analysis, parasites were cloned from a long-term, continuous culture of the IT4/25/5 clone A4 (Figure 1) [6]. The IT4/25/5 (IT4) parasite genotype is unusual because the parasite maintained its cytoadhesion capabilities after *in vitro* adaptation [50], [51], making it a primary model for this virulence determinant. A set of 54 *var* genes has been reported from the IT4 parasite genotype [9], [10]. The A4 cloned parasite line expresses a *var* gene (*A4var/IT4var14*) that has an unusually high switch frequency (∼1–2% per generation) [6], [52], resulting in PfEMP1 heterogeneity at the population level in the long term culture. After 70 parasite divisions in continuous culture, the long-term A4 culture had completely switched away from the *A4var* gene (*IT4var14*) and expressed a mixture of different *var* genes at low levels with *IT4var26*, *IT4var31*, and *IT4var37* predominating (Figure 2). Both *IT4var31* (previously referred to as *C18var*) and *IT4var37* (previously referred to as *AFBR6)* were also found to be common switch events in two previous studies of *var* gene switching within the A4 parasite lineage [7], [52], suggesting that these particular genes may have high “on” rates in unselected cultures.

**Figure 3:**
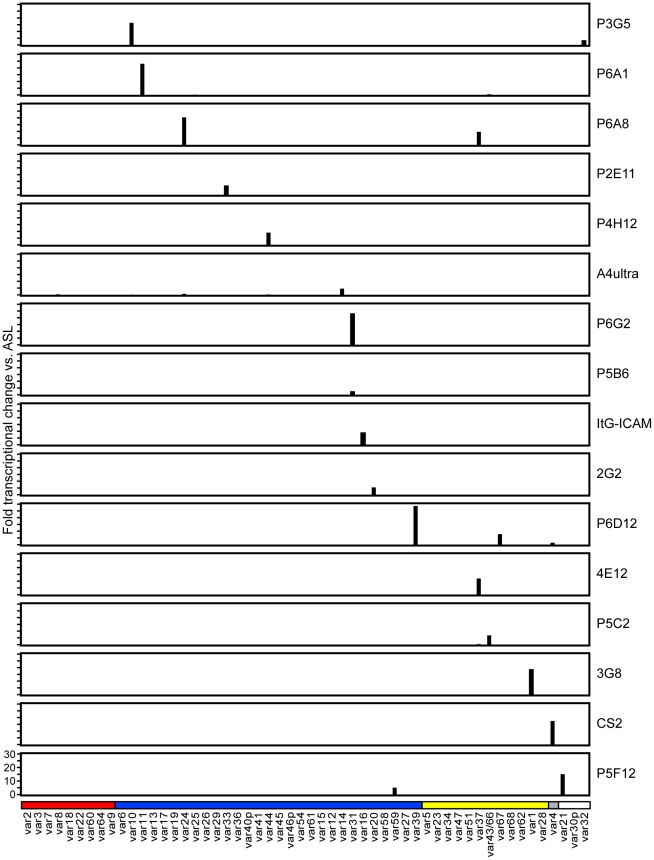
Analysis of *var* gene transcription in parasite lines at the time of infected erythrocyte binding assays. RNA was harvested from ring stage parasites in the same cycle that infected erythrocyte binding assays were performed, and *var* expression profiling was performed by qRT-PCR. Most parasite lines expressed a single dominant *var* gene transcript, and some parasite lines had a secondary *var* transcript. Other *var* genes were expressed at negligible levels. Gene expression was normalized to the housekeeping control gene adenylosuccinate lyase (*asl*). The scale of the Y-axis is the same for all parasite lines. The A4 primer set is arranged along the *x*-axis by Ups category; UpsA (red), UpsB (blue), UpsC, (yellow), and UpsE (gray) groups, as well as three genes for which the Ups type has not been determined (white). The name of each parasite line is indicated at the right of its respective transcription profile.

Initially, 17 subclones were isolated from the long-term A4 parasite culture by limited dilution cloning (Figure 1). From *var* transcription profiling, 6 of the subclones transcribed *IT4var31* as either the primary or secondary *var* transcript, 8 transcribed dominant *var* gene transcripts distinct from each other, and a dominant *var* transcript (present at greater than 50% of the total *var* transcripts) could not be identified in 3 of the subclones by qRT-PCR analysis (Table 1, and data not shown). Ten subclones that primarily expressed single dominant *var* transcripts, including two that expressed *IT4var31*, were selected for phenotypic analysis (Figure 3).

**Table 1:** Phenotypic and *va*r transcriptional profile of parasite panel.

			qRT-PCR initial typing^c^		qRT-PCR: binding^d^	
Parasite	Gelatin^a^	kahrp^b^	major transcript	% transcripts	major transcript	% transcripts
R29	-	+	nd	nd	nd	nd
Pf13	-	+	nd	nd	nd	nd
VarO	-	+	nd	nd	nd	nd
CS2	+	+	nd	nd	var4	99%
A4ultra	+	+	nd	nd	var14; var24	25%;6%
P6D12	+	+	var39; var67	60%; 36%	var39; var67	74%;21%
P6A1	+	+	var11	92%	var11	81%
P4H12	+	+	var44	100%	var44	100%
P2E11	+	+	var33	90%	var33	96%
ItG-ICAM-1	+	+	nd	nd	var16	60%
P6G2	+	+	var29;var31	57%;42%	var31	97%
P5B6	+	+	var31	100%	var31	89%
P5C2	+	+	var43/var66;var31	71%;19%	var43/66	78%
3G8	+	+	var1	99%	var1	98%
P6A8	+	+	var24;var37	79%;16%	var24;var37	66%;32%
4E12	+	+	var37	94%	var37	95%
P5F12	+	+	var21;var59	70%;30%	var21;var59	73%;27%
P3G5	+	+	var10;var32	52%;48%	var10;var32	80%;19%
2G2	-	-	var20	88%	var20	96%

a The ability of infected erythrocytes to float in 0.7% gelatin was used as a surrogate for knob positivity.

b Indicates whether the knob-associated histidine rich protein gene could be amplified from cDNA.

c Profiling of *var* transcription was performed after initial parasite expansion following limited dilution cloning. Transcripts representing greater than 5% of the total *var* messages are listed.

d Profiling of *var* transcription was repeated at the time of infected erythrocyte binding assays as above. nd - not done.

Of interest, there was negligible UpsA transcription in the long-term A4 culture (Figure 2), and none of the isolated subclones expressed an UpsA *var* gene (Figure 3). To attempt to enrich for UpsA variants, the long-term A4 culture was panned on CD36 receptor protein and non-adherent parasites were selected. Although the *var* transcriptional profile was modified after CD36 negative selection, this approach did not enrich for UpsA variants. Instead, the frequent switch variant *IT4var31* was the resulting major transcript (data not shown). This again indicates that UpsA genes are rare switch events in long-term A4 cultures.

**Figure 4:**
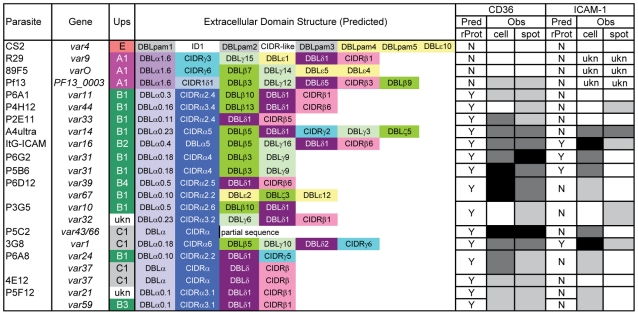
Schematic representation of *var* transcription in the parasite panel and summary binding to CD36 and ICAM-1 receptor proteins. Parasite name, predominant *var* transcript(s) by qRT-PCR, Ups categorization, and predicted protein architecture are shown for parasite lines included in the panel, with the exception of the negative binding control line (2G2). Predicted binding of native PfEMP1 to CD36 and ICAM-1 receptor proteins based on previous analysis of CIDR::CD36 [46] and DBLβ::ICAM-1 interactions [44] are shown, along with observed binding to these receptor proteins in both cell and recombinant protein platforms. Low, moderate, and high binding levels are indicated in gray-scale: clear = <15 IE per 100 CHO cells, <80 IE per mm^2^; light gray = 16–50 IE per 100 CHO cells, 81–500 IE per mm^2^; dark gray = 51–100 IE per 100 CHO cells, 501–1000 IE per mm^2^; black = >101 IE per 100 CHO cells, >1000 IE per mm^2^. Ukn means the Ups type or binding behavior is unknown.

To create a more representative panel for phenotypic analysis, six previously isolated parasite lines from the IT4/FCR3 strain and three UpsA parasite lines from different parasite strains (IT4/FCR3, Palo Alto 89F5, and 3D7) (Figure 1) were included in the binding studies. The three UpsA parasite lines (R29, VarO, and Pf13) were isolated by rosette enrichment and selected for high purity using specific monoclonal antibodies to the respective PfEMP1 proteins [53]. Altogether, 19 parasite lines were examined representing all three major *var* gene groups. Three of the parasites in the panel expressed an UpsA protein as the dominant *var* transcript, ten expressed an UpsB *var* gene, three expressed an UpsC *var*, one expressed the unique UpsE linked transcript (*IT4var4*, *var2CSA*), and for one parasite, the Ups category of the dominant *var* transcript has yet to be determined (Figure 4). The remaining parasite in the panel, 2G2, is knobless and was employed as a negative binding control (Table 1) [54]. Most parasites in the panel expressed distinct dominant *var* transcripts, except two subclones (P6G2 and P5B6) expressed *IT4var31,* and two others (P6A8 and 4E12) expressed *IT4var37/AFBR6* as either the dominant or secondary *var* transcript (Figure 3 and Table 1).

To confirm the presence of knobs on the IE surface, which are known to be important in PfEMP1 anchoring and infected erythrocyte binding [41], [54], [55], parasites were tested for transcription of the knob associated histidine rich protein (*kahrp* ^+^) by RT-PCR and floated by gelatin sedimentation (gelatin^+^). All parasites in the panel were positive in both assays, except for the negative control 2G2 parasite line, which lacks *kahrp* and therefore sedimented in gelatin. In addition, the three rosette-forming UpsA parasites all transcribed *kahrp* but sedimented in gelatin because they were originally isolated on the basis of their property to sediment in Ficoll (Table 1). To confirm the identity of *var* gene transcription at the time of binding assays, RNA was harvested within the same growth cycle that binding assays were performed. For these assays, thawed parasite stabilates were grown for 4 to 5 cycles to generate sufficient parasite material, and parasites were generally analyzed a total of 18–20 cycles from initial parasite cloning. In general, the dominant *var* transcript did not change between the initial qRT-PCR characterization performed after limited dilution cloning and the second round of -typing done at the binding assay (Table 1). In only one parasite line, P6G2, the previous dominant transcript was replaced by the secondary *var* transcript that was present before freezing (Table 1). At the time of the binding analysis, the average fold transcription of dominant *var* transcripts relative to the *asl* housekeeping gene was 14.2 (range 2.8–28.1). Furthermore, most parasite lines were significantly enriched for a single predominant *var* transcript (Figure 3), and only 8 parasite lines contained a secondary *var* transcript at greater than 5% of the total *var* transcripts (Table 1). In most cases, the secondary transcript was present at much lower levels than the dominant *var* transcript. Thus, *var* gene transcription was stable over the short-term culture period used to perform these assays. For the three UpsA variants, PfEMP1 expression was established by flow cytometry with specific monoclonal antibodies to be 79% or higher using conservative gating criteria (Figure S1). Furthermore, all three lines formed rosettes in O-type RBCs: R29 (rosetting rate = 37%, 89% mAb reactivity R29), VarO (rosetting rate = 73%, 79% mAb reactivity VarO), Pf13 (rosetting rate = 52%, 94% mAb reactivity Pf13_0003). Therefore, all of the parasites in the panel were highly homogenous for one or two *var* transcripts, and UpsA parasite lines were highly pure for a single expressed PfEMP1 variant.

### CD36 binding of infected erythrocytes is highly predicted by the type of CIDR domain in PfEMP1 proteins

To investigate whether infected erythrocyte binding to CD36 could be predicted from sequence information and binding studies of isolated CIDR domains [46], the complete panel of parasite lines was analyzed for binding to both CHO745-CD36 and immobilized CD36 recombinant protein. Because rosettes of uninfected red blood cells can interfere with binding or make bound IEs more susceptible to disruption during washing stages, the rosettes of the three UpsA variants were first disrupted using heparin sulfate prior to binding analysis. Previous work has shown that sulfated glycoconjugates can enhance binding to CD36 on cell surfaces [56]. Therefore, as a control for the three rosetting parasite lines, all of the parasites in the panel were treated with heparin sulfate and tested for binding to immobilized CD36 recombinant protein. Heparin sulfate treatment greatly diminished rosette formation in the three UpsA parasite lines (<10%) (Figure S2), but had minimal effect on infected erythrocyte binding to immobilized CD36 recombinant protein. Overall, parasites had comparable binding levels in the presence or absence of heparin sulfate (Figure S2). In addition, two non-rosetting, CD36 binding parasite lines (A4ultra and ItG-ICAM-1) were tested for binding to CHO745-CD36 cells in the presence or absence of heparin sulfate. Similar to what has been reported previously [56], sulfated glycoconjugates increased IE binding to CHO745-CD36 (Figure S3). Because heparin sulfate may slightly enhance IE adhesion to CHO-CD36 and did not modify IE adhesion to immobilized CD36, the binding assay was then repeated for all of the non-UpsA parasites in the absence of sulfated glycoconjugates. In contrast, binding of the three UpsA lines to CHO-CD36 and immobilized CD36 was repeated in the presence of sulfated glyconjugates to prevent infected erythrocyte rosetting interfering with the binding results.

**Figure 5:**
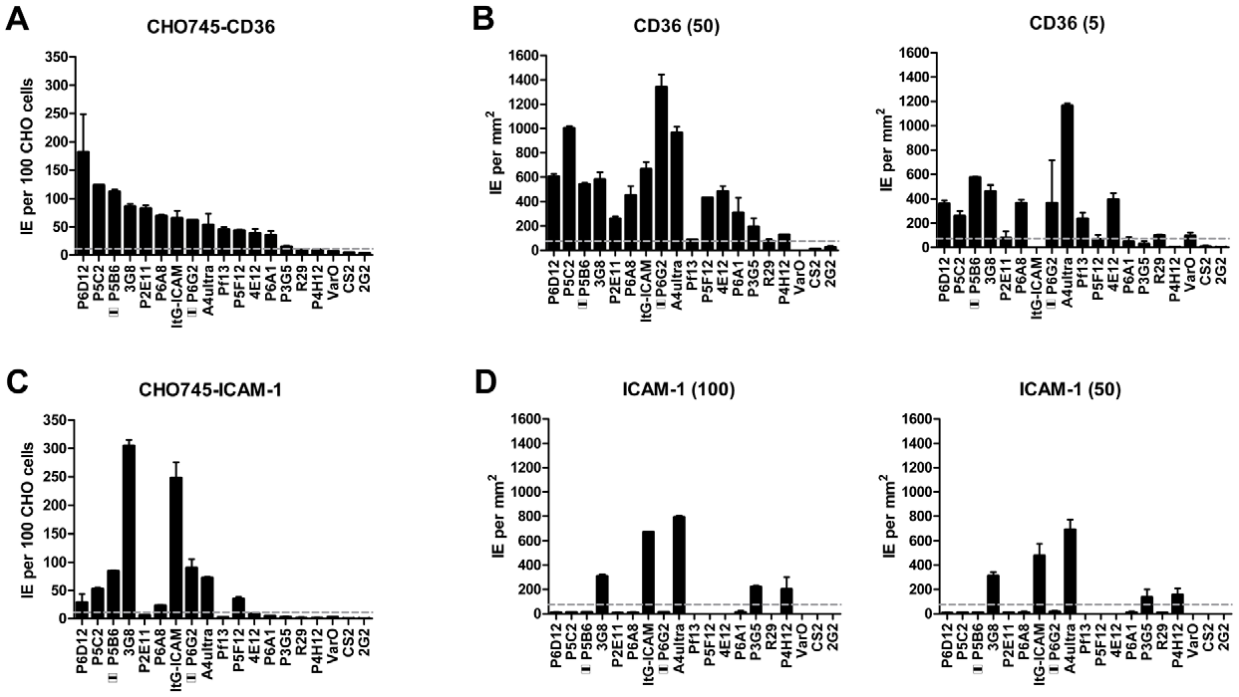
Infected erythrocyte binding to CD36 and ICAM-1. Parasites in the panel were assessed for binding to transfected CHO745 cell lines or recombinant proteins. (**A**) Infected erythrocyte binding to CHO745 cell transfectants expressing human CD36 receptor. (**B**) Infected erythrocyte binding to recombinant CD36-Fc protein immobilized on polypropylene substrate at 50 µg/mL and 5 µg/mL concentrations. (**C**) Infected erythrocyte binding to CHO cell transfectants expressing human ICAM-1 receptor. (**D**) Infected erythrocyte binding to recombinant ICAM-1-Fc protein immobilized on polypropylene substrate at 100 µg/mL and 50 µg/mL. The two parasite lines that express *IT4var31* as the predominant *var* gene are indicated by boxes with horizontal bars. An arbitrary threshold for positive binding (grey dashed line) was calculated as the mean level of infected erythrocyte binding plus two standard deviations either to untransfected CHO745 cells or to spots containing 2% bovine serum albumin, respectively. Error bars represent the range of binding between two replicate experiments.

**Figure 6:**
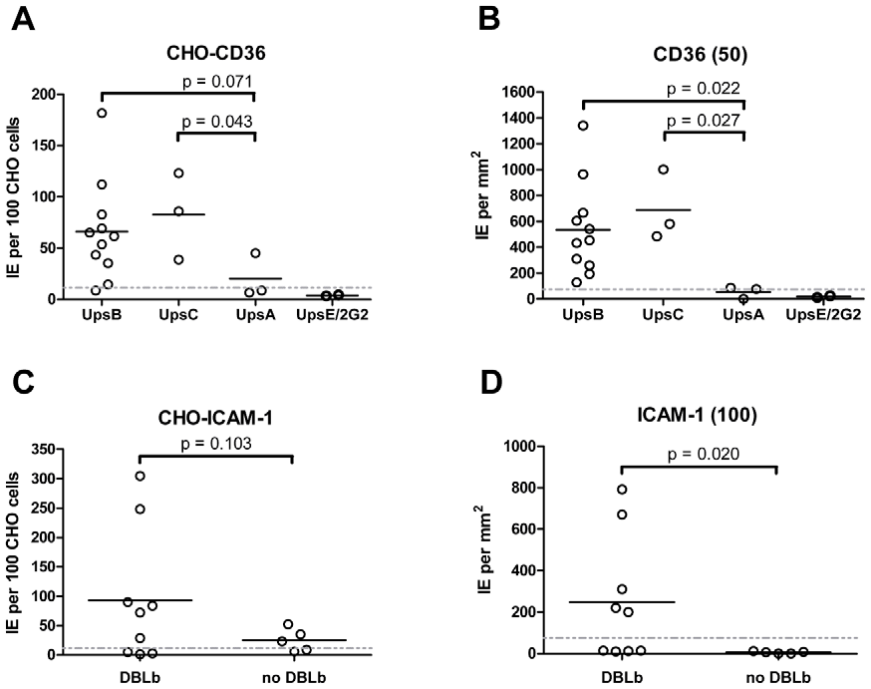
Infected erythrocyte binding to CD36 and ICAM-1. Dot plots show averaged results of replicate infected erythrocyte binding experiments for individual cloned parasites lines contained in the panel (Figure 5) and grouped either by (A, B) Ups classification of major *var* transcript or (C, D) presence of DBLβ adhesion domain in the major *var* transcript. Mean infected erythrocyte binding was compared between groups using non-paired, 1-tailed t-tests. P-values are indicated according to the 95% confidence interval. An arbitrary threshold for positive binding (grey dashed line) was calculated as the mean level of infected erythrocyte binding plus two standard deviations either to untransfected CHO745 cells or to spots containing 2% bovine serum albumin, respectively. (**A**) Infected erythrocyte binding to CHO745-CD36. (**B**) Infected erythrocyte binding to recombinant CD36-Fc fusion protein immobilized in 10 µL spots at 50 µg/mL onto polystyrene substrate. (**C**) Infected erythrocyte binding to CHO745-ICAM-1. (**D)** Infected erythrocyte binding to recombinant ICAM-1-Fc fusion protein immobilized in 10 µL spots at 100 µg/mL onto polystyrene substrate.

Overall, there was a significant correlation between CHO745-CD36 and spotted CD36 protein formats (Figure 5, Spearman's Rho = 0.75, p<0.001). Although the level of CD36 binding varied between parasite lines, most of the parasites bound CD36, with the exception of UpsA/E groups (Figure 6). The three UpsA parasites were at the lower spectrum of CD36 binding in both cell and recombinant protein binding assays, and were basically indistinguishable from the negative control, knobless parasite line, and the UpsE parasite line that does not bind CD36 (Figure 6). Furthermore, CD36 binding was highly predicted by the type of CIDR1 domain in the PfEMP1 head structure (Figure 4). Indeed, only two parasites in the panel that were predicted to bind CD36 did not bind to CHO-CD36 cells. However, both exceptions (P4H12 and P3G5) bound at a low level to 50 µg/mL rCD36, but not to 5 µg/mL rCD36 (Figure 5), and therefore may be lower affinity CD36 binders. In group-wide comparisons, UpsB and UpsC had a higher mean CD36 binding level than UpsA. This difference was significantly different in the immobilized CD36 binding assay and between the UpsC and UpsA groups in the CHO-CD36 assay, and just missed significance between the UpsB and UpsA groups in the CHO-CD36 assay (Figure 6). Taken together, infected erythrocyte binding was highly predictable based on the type of CIDR domain (Figure 4), and the UpsA group appears to be under less selection to bind CD36.

### ICAM-1 binding was strongly associated with larger, DBLβ containing PfEMP1 proteins

To test whether ICAM-1 binding was associated with larger PfEMP1 proteins containing DBLβ domains [44], the parasite panel was analyzed for binding to CHO745-ICAM-1 and recombinant ICAM-1 protein. Again, to prevent rosettes from interfering with the binding analysis, the three UpsA parasite lines were treated with sulfated glycoconjugates prior to binding analysis, and as a control, two non-rosetting, ICAM-1 binding parasite lines (A4ultra and ItG-ICAM-1) were tested for ICAM-1 binding in the presence or absence of sulfated glycoconjugates. Sulfated glyconjugates reduced binding of A4ultra in the CHO745-ICAM-1 assay and binding of both parasite lines to spotted ICAM-1 recombinant protein (Figure S3), similar to what has been reported before [56]. Because of the potential for sulfated glyconjugates to interfere with ICAM-1 binding in the cell and recombinant protein assays, the three UpsA parasite lines were not considered in the ICAM-1 binding analysis.

In the cell binding assay, two parasite lines bound at a high level (>2 IEs/CHO745-ICAM-1), three bound at moderate level (0.5–2 IEs/CHO745-ICAM-1), and the remaining parasite lines bound at a low level or did not bind ICAM-1 (Figure 5). While there was good consistency between the cell and recombinant protein assays for the two high level ICAM-1 binders, there was more discordance for weaker ICAM-1 binders (Figure 5). Only three parasite lines bound ICAM-1 in both platforms (3G8, ItG-ICAM-1, and A4ultra), and two parasite lines that bound at a moderate level to CHO745-ICAM-1 did not bind to immobilized ICAM-1 protein (Figure 5). Notably, both parasite lines express the *IT4var31* transcript, which has been suggested to be a weaker ICAM-1 binding variant that is trypsin-resistant [57], [58]. To confirm whether binding was trypsin-resistant, P5B6-infected erythrocytes expressing *IT4var31* were treated with 1 mg/mL trypsin prior to ICAM-1 binding analysis. Trypsin treatment reduced CD36 binding and increased binding to recombinant ICAM-1 (Figure S4), and therefore may have cleaved or truncated the PfEMP1 head structure. The increase in ICAM-1 binding could be blocked by anti-ICAM-1 antibody (mAb 15.2) and not by anti-CD36 isotype control antibody (FA6-152) (Figure S4). In contrast, identical trypsin treatment of 3G8 (*IT4var1*) and ItG-ICAM-1 parasite lines (*IT4var16*) abolished binding to both CD36 and ICAM-1 (data not shown). Thus, as predicted from binding of the isolated DBLβ domain [58], IT4var31 was associated with ICAM-1 binding, but the cell binding assay was more sensitive than immobilized protein in detecting this interaction. Two of the parasite lines also bound at a low level to immobilized ICAM-1 recombinant protein but did not bind CHO745-ICAM-1. Thus, there may be differences in the sensitivity of the two platforms to detect lower affinity ICAM-1 interactions, or some of the low level binding interactions may not have been specific.

**Figure 7:**
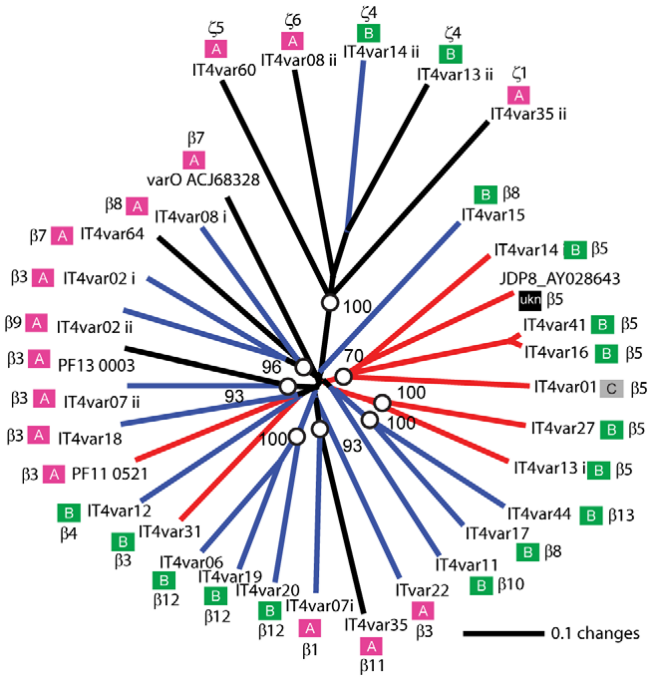
Phylogentic comparison of DBLβ domains that bind or do not bind ICAM-1. A tree of DBLβ sequences was constructed using new domain boundaries [10] and was used to reanalyze the DBLβ-ICAM-1 interaction. Bootstrap values over 70% are indicated with open circles. Recombinant DBLβ proteins from the IT4 parasite genotype that were previously shown to bind ICAM-1 are indicated with red lines, non-binding sequences have blue lines, and untested sequences are indicated with black lines [46]. Two ICAM-1 binding sequences from the 3D7 and JD8 parasite isolates are also included [45], [59]. Ups A–C grouping is indicated next to the gene name, as well as the DBL subclassification according to Rask et al [10].

Overall, ICAM-1 binding was strongly associated with larger PfEMP1 proteins that contained a DBLβ domain. Seven of the ten parasites lines that expressed a dominant *var* transcript containing a DBLβ domain bound to ICAM-1 in either the cell or recombinant protein platform (Figure 4), and parasite lines without a DBLβ either bound extremely weakly or did not bind ICAM-1 (Figure 6). This difference was significant in the immobilized ICAM-1 assays (1-tailed t-test, p = 0.020) and just missed significance in the CHO745-ICAM-1 assay (1-tailed t-test, p = 0.103). Recently, there has been a reclassification of DBL and CIDR domains into additional subtypes based on a comparison of 7 parasite genomes in which DBLβ domains were subclassified into 13 sub-types [10]. Of interest, all three parasites that bound in both the CHO-ICAM-1 and immobilized ICAM-1 assays expressed a DBLβ5 domain (Figure 4). To investigate if DBLβ5 could be a marker for ICAM-1 binding, we reanalyzed the recombinant DBLβ-ICAM-1 binding data [44]. In the IT4 parasite genotype, 7 of 23 DBLβ domains bound ICAM-1. Of the 7 ICAM-1 binders, 6 were DBLβ5 sequences, and there were no DBLβ5 domains that did not bind ICAM-1 (Figure 7). Significantly, an ICAM-1 binding parasite from India (JDP8-ICAM-1, AY028643) [59] also uses a DBLβ5 domain to bind ICAM-1 (Figure 7). The fact that ICAM-1 binding was 100% predictable in the IT4 parasite genotype, and that a different parasite isolate from India also uses DBLβ5 for binding, strongly supports this domain as a marker for ICAM-1 binding. There are also two DBLβ3 sequences that bound ICAM-1, one from the IT4 parasite genotype and one from the 3D7 parasite genotype [45], but several other DBLβ3 sequences did not bind ICAM-1 as recombinant proteins (Figure 7). Taken together, ICAM-1 binding was strongly associated with the DBLβ domain, and the DBLβ5 marks a category of larger PfEMP1 variants that encode this adhesion property.

### Infected erythrocyte binding to additional receptors was rare

**Figure 8:**
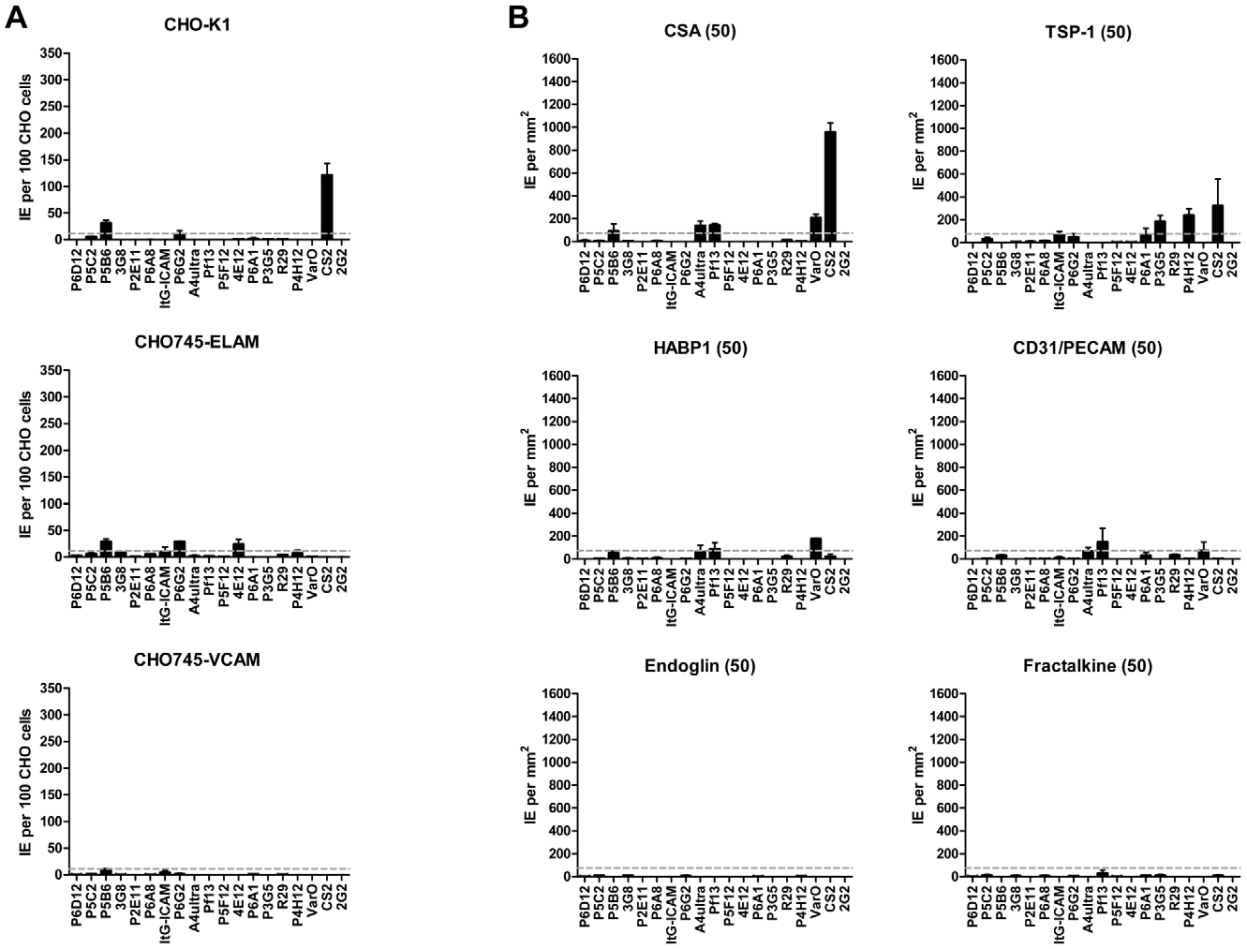
Infected erythrocyte binding to other candidate cytoadhesion receptors. Parasites in the panel were assessed for binding to transfected CHO745 cell lines or recombinant proteins. (**A**) Infected erythrocyte binding to CHO cells expressing chondroitin sulfate A (CHO-K1), E-selectin (CHO745-ELAM-1), or vascular leukocyte adhesion molecule 1 (CHO745-VCAM-1). (**B**) Infected erythrocyte binding to immobilized chondroitin sulfate A (CSA) and the recombinant proteins TSP-1, HABP1, CD31, endoglin, and fractalkine. All proteins and CSA were immobilized on polystyrene substrate at 50 µg/mL. Infected erythrocyte binding to transfected cell lines were corrected by subtracting background binding to untransfected CHO-745 cells. Binding to recombinant proteins was corrected by substracting binding to bovine serum albumin, which was employed as the blocking agent in all assays. An arbitrary threshold for positive binding (grey dashed line) was calculated as the mean level of infected erythrocyte binding plus two standard deviations either to untransfected CHO745 cells or to spots containing 2% bovine serum albumin, respectively. Error bars represent the range of binding between two replicate experiments.

Infected erythrocytes have been reported to bind a number of host receptors [20], but for the most part binding has only been tested on one or a few parasite lines. Using transfected cells or recombinant proteins, the 19 parasite lines were assayed against 8 additional receptors: Endothelial Leukocyte Adhesion Molecule 1 (E-selectin), Vascular Cell Adhesion Molecule 1 (VCAM-1), CHO receptor “X”, Hyaluronan Binding Protein 1 (HABP1), Platelet Endothelial Cell Adhesion Molecule-1 (CD31/PECAM-1), Thrombospondin-1 (TSP-1), CSA, and Fractalkine. Whereas a few parasite lines bound at a low level to TSP-1 and CHO-ELAM-1, there was negligible binding to most receptors tested (Figure 8). Two of the UpsA parasites (Pf13 and VarO) bound at a low level to HABP1, CD31, and CSA. However, binding of UpsA parasites was performed in the presence of sulfated glycoconjugates to disrupt rosettes, and they also had higher background binding to bovine serum albumin (BSA) employed as a blocking agent for binding assays (Figure 8, and data not shown). As expected, the strongest CSA-binder in the panel was the CS2 parasite line in both the CHO-K1 cell and CSA spot formats (Figure 8). CS2 expresses the VAR2CSA PfEMP1 protein that has been shown to be the primary PfEMP1 variant associated with CSA binding [60], [61]. Most of the other receptors tested did not support strong adhesion of infected erythrocytes in these binding assays and it is questionable whether all of the observed weak interactions are physiologically relevant.

## Discussion

PfEMP1 proteins/*var* genes are classified into three main subfamilies (UpsA, UpsB, and UpsC) that have different host expression profiles [35]–[37], [39]. Both binding strength and specificity of IEs are likely to influence disease severity during an infection; therefore, it is important to understand whether PfEMP1 subfamilies have evolved specialized properties for distinct host/biological niches. Studies of malaria during pregnancy have demonstrated how a specific PfEMP1 variant can precipitate severe disease in otherwise immune women by altering IE tropism for the placenta [14], [29], [62]. Although VAR2CSA appears to be unique in its ability to confer high-affinity binding to CSA in the placenta [60], [61], [63], it offers a paradigm for the role of specific PfEMP1s in disease. UpsA classified PfEMP1 proteins are frequently observed in young children with limited anti-malaria immunity or experiencing severe malaria [35]–[39]. Unlike VAR2CSA, the adherence characteristics of UpsA proteins are poorly understood and limited largely to predictions of binding based on studies of isolated adhesion domains [44]–[46]. To investigate a correlation of PfEMP1 binding specificities with disease outcome, the binding characteristics of at least a representative sample of the three main subgroups (UpsA, UpsB and UpsC) have to be known. In this study, we employed a panel of different PfEMP1 types to test binding predictions based upon studies of single PfEMP1 domains.

While UpsA variants appear to be commonly expressed in early childhood infections and non-immune individuals [35]–[39], very little is known about what may account for this preferential expression in the malaria naïve. Investigation is hampered because most *P. falciparum* infections contain a mixture of PfEMP1 variants and even minor parasite subsets may obscure binding analysis. In addition, gene silencing of UpsA variants has been observed upon *in vitro* adaptation [64]. In long term *in vitro* adapted parasite cultures grown without selection for specific *var* gene expression, UpsA variants were expressed at a low level, and an UpsB (*IT4var31/C18var*) and an UpsC (*IT4var37*/*AFBR6*) *var* gene appeared to be the most common switch events. Both were also found to be frequently activated in previous clonal analyses in this strain background [7], [52] and thus may have a higher “on” rate under *in vitro* culture conditions. One study found that *var* genes in central chromosome regions had lower switch rates than those in telomeric regions [65], but inherent differences were not consistently observed in a different parasite line [52]. The chromosome positions of *IT4var31* (UpsB) and *IT4var37* (UpsC) have not been mapped and therefore we cannot comment on whether this observation held true in our study or not. However, our findings indicate that promoter type is not the main determinant of *var* gene “on” rate as far as UpsB and UpsC type *var* genes are concerned. In the case of UpsA variants, the promoter type did seem to determine *var* gene expression rate by significantly reducing it. To overcome these problems, we used specific monoclonal antibodies to generate three distinct UpsA parasite lines of high purity for the parasite panel.

In epidemiological studies, CD36 and ICAM-1 binding are the most common adhesion traits in the parasite population [17], [19], but their distribution among different members of the PfEMP1 family is only partially understood [44]–[46], [58], [66]. In the parasite panel, CD36 was by far the most common binding partner, followed by ICAM-1 and TSP-1. CD36-binding was nearly 100% predictable and was always associated with a CIDRα type domain in the protein head structure, while the three UpsA variants had different sequence types (CIDRγ and CIDRδ) and did not bind CD36 or only bound at a low level. Thus, in the absence of a CIDRα domain, other potential CD36 ligands [67], [68] were unable to compensate for infected erythrocyte binding. Moreover, the level of CD36 binding differed between isogenic parasites expressing different PfEMP1 variants, suggesting that PfEMP1 sequence variability or surface expression levels have an important role in influencing the overall binding affinity of infected erythrocytes.

The UpsA group contains three different types of CIDR1 sequences (α1, γ, or δ) [10], [12], [40], [46]. Although the three UpsA parasites in the panel were all selected for rosetting, “rosetting” and “non-CD36 binding” can exist as independent phenotypes. For instance, the non-CD36 binding CIDR domains identified in this study may potentially be found in non-rosetting group A genes, and there is evidence that CD36 is able to act as a host receptor for rosetting in the Malayan Camp parasite strain and some field isolates [69]. This parasite panel did not contain any representation of the CIDRα1 subtype, which is found in approximately half of UpsA proteins [10]. However, it has previously been shown that recombinant CIDRα1 subtype domains do not bind CD36 [46], and CD36 selection led to loss of expression of an UpsA gene in a mixed parasite culture that expressed a CIDRα1 subtype [70]. Taken together, the results suggest the UpsA group is not under strong selection for CD36 binding, and it will be interesting to determine if the UpsA protein head structure is selected for specific binding properties that support microvasculature sequestration by a mechanism different from CD36 binding. Part of this selection may be for infected erythrocyte rosetting [71], [72], but the UpsA group may encode other adhesion properties [47].

After CD36, ICAM-1 is one of the most common adhesion properties, and the two receptors synergize to mediate infected erythrocyte binding under flow [22], [23]. ICAM-1 is upregulated on brain endothelium during malaria infections and has been proposed to be a potential cerebral sequestration receptor [24]. ICAM-1 binding has previously been mapped to the DBLβ domain [44], [45], [58], [59], [73]. Our study confirms this association as the DBLβ5 domain was 100% associated with ICAM-1 binding in both parasite lines and recombinant proteins. It also shows that not all DBLβ domains bind to ICAM-1. In future work using patient samples it may be interesting to investigate how well transcription of *var* genes containing a DBLβ5 domain can predict ICAM-1 binding. Overall, this study identifies a category of large UpsB and UpsC PfEMP1 containing CIDRα and DBLβ5 subtype domains that were 100% associated with CD36 and ICAM-1 binding. In a comparison of *var* gene repertoires from 7 parasite strains, the CIDRα and DBLβ5 domains were always found together in tandem arrangement (27 of 399 full or partial length *var* genes), and the DBLβ5 domain was never associated with a predicted “non-CD36 binding” CIDR domain. This suggests the association has not evolved by chance and that the CIDRα-DBLβ5 domain combination may be under dual selection for binding to CD36 and ICAM-1. Both receptors are co-displayed on many of the same cell types (endothelial, monocyte, and dendritic cells) and may provide the parasite opportunities to manipulate host cells [74], [75], thus contributing to their strong selection in the PfEMP1 repertoire. There were also a few DBLβ3 domains that bound to ICAM-1, but these were found in association with both CD36 binding and non-CD36 binding CIDR domains. Thus, CD36 and ICAM-1 have left strong signatures of selection detectable by PfEMP1 adhesion domain sequence classification, despite the extensive sequence diversity in the family.

Other PfEMP1 adhesion properties examined appear to be much rarer or may only play an additive role in overall binding affinity. Nearly all PfEMP1 proteins have four or more extracellular domains. In addition to undefined binding properties, other PfEMP1 domains may also function as “spacers” to position the PfEMP1 head structure and adjacent DBLβ away from the IE surface in order to engage CD36 and ICAM-1 [76]. A potential caveat is that binding was performed under static adhesion conditions, and individual host recombinant proteins were employed in the protein binding assays. However, all host receptors examined were originally defined under similar static adhesion conditions. Furthermore, static adhesion assays are capable of detecting host receptor interactions that support both rolling (ICAM-1, TSP-1) and stationary (CD36) cytoadhesion of infected erythrocytes under flow conditions [21]. Cooperative binding is likely necessary to mediate firm adhesion under flow [21]–[23], but from this analysis CD36 binding is under greatest selection and contributes the greatest binding avidity in different PfEMP1 proteins.

These results reveal a fundamental difference in CD36 binding between Ups groups that has important implications for how parasites establish infections in individuals of varying levels of immunity. UpsA proteins are more commonly expressed in children with low immunity [35], [36], [39]. Later, as malaria immunity develops, it may be significant that the proportion of non-UpsA types and CD36 binding variants increases. It is interesting to speculate that non-CD36-binding parasites may experience a selective advantage over their CD36-binding counterparts in patients with limited exposure to malaria. CD36-binding parasites are thought to manipulate both host innate and adaptive immune responses by interacting with monocytes and dendritic cells [74], [75], [77], [78]. In the malaria naïve, these interactions may be less important, or UpsA variants may possess other advantages or means of host manipulation. While UpsA variants have not been clearly associated with disease in all studies [79], they are more abundant in patients with severe malaria [80], [81] and have been associated with cerebral malaria infections in children in Mali [38]. A greater proportion of UpsA variants in early infections could potentially contribute to why CD36 binding levels are very low in children with severe malaria anemia [17], [19], or these variants could alter the pattern of sequestration to microvascular beds, such as brain endothelium, where CD36 binding levels are extremely low [24]. Therefore, it will be important to learn more about this group of proteins.

In conclusion, the PfEMP1 protein family has diversified under dual selection to evade host immunity and mediate infected erythrocyte binding. The development of a parasite panel enriched for distinct PfEMP1 expression from the major Ups groups has facilitated the testing of binding predictions, and may have potential applications for investigating immune acquisition to the family of proteins. This comparative analysis demonstrates the predictability of *P. falciparum*-IE binding to the two major cytoadhesion receptors CD36 and ICAM-1 and provides new insight into how natural selection may be shaping the PfEMP1 binding repertoire to exploit distinct host niches of varying anti-malaria immunity.

## Materials and Methods

### Ethics statement

Human blood was used for *P. falciparum* culture in this study. Donor blood was obtained from healthy volunteers under a minimal risk, standardized, Institute protocol (protocol number HS013) that was approved by the Western Institutional Review Board. Written informed consent was obtained from all blood donor study participants.

### *P. falciparum* culture conditions

The three UpsA variants were isolated by gelatin sedimentation followed by positive selection with specific monoclonal antibodies against the respective NTS-DBLα domain. The VarO parasite clone was generated from the Palo Alto strain as described by rosette enrichment and selection with monoclonal antibody D15–50 [82]. The R29 parasite (IT4 parasite strain) has been described previously [6], [7], [83]. Highly enriched parasite cultures expressing the R29 PfEMP1 protein and Pf13 (3D7 strain) were isolated by similar methodologies to the VarO parasite line using rosette enrichment and specific monoclonal antibodies against the R29-DBLα domain (3B13C5) or the Pf13_0003-DBLα domain (J3.21) [53]. The ItG-ICAM-1 parasite line was derived by ICAM-1 selection [18], CS2 by CSA selection [84], and the 3G8, 4E12, and 2G2 parasite lines by limited dilution cloning [52]. The remaining parasite lines were derived from IT4/25/5 clone A4 [6] by limited dilution cloning. Infected erythrocytes were cultured under standard conditions using human O red blood cells (RBCs) in RPMI-1640 medium (Invitrogen) supplemented with 10% pooled human A^+^ serum and an atmosphere of 5% CO_2_, 5% O_2_, and 95% N_2_ at 37°C. Synchronization of parasite growth was achieved by treatment with 5% sorbitol in PBS. Gelatin sedimentation assays were performed in RPMI-1640 medium containing 0.7% porcine gelatin (Sigma) for 45 minutes at 37°C. Enrichment of infected erythrocytes (IE) in the gelatin supernatant was determined by counting >300 methanol-fixed, Giemsa-stained RBCs under 1000X magnification. Rosette formation was visualized after infected red blood cell nuclei were stained by ethidium bromide. The rosetting rate was calculated by determining the percentage of rosette-forming infected cells in the mature parasite population.

### Chinese hamster ovary cell culture conditions

CHO-K1, CHO745, and CHO745 transfectants expressing CD36, ICAM-1, E-selectin, or VCAM-1 were cultured in F-12 Kaighn's medium supplemented with 10% fetal calf serum and 0.5 mg/mL geneticin (Gibco). The CHO745 transfectants were described in Buffet et al. [85]. Recombinant protein surface expression was monitored by flow cytometry on a monthly basis using receptor-specific monoclonal antibodies (R&D Systems), and cells were replaced if the percentage of transfected cells or mean fluorescence intensity diminished by greater than 20%.

### Limited dilution cloning of parasite lines

An A4 parasite clonal line [6] was grown continuously under standard conditions for more than 70 growth cycles in the absence of overt selection. IEs were periodically enriched for knob expression by floatation in 0.7% porcine gelatin (Sigma) dissolved in RPMI-1640 (Invitrogen) at 37°C. Prior to limited dilution cloning, RNA was collected and a profile of *var* transcription was determined by quantitative real-time polymerase chain reaction (qRT-PCR) using a primer set designed to amplify unique sequence tags within the repertoire of IT4 *var* genes [86]. Individual infected erythrocytes were obtained on two separate occasions by limited dilution cloning after more than 78 and 84 cycles of continuous parasite growth, respectively, at a seeding rate of 0.5 infected erythrocytes per well. Initial frozen stabilates were collected after approximately 14–15 cycles of growth and parasite lines were typed for *var* gene expression by qRT-PCR.

### Determination of *var* transcription by qRT-PCR

The determination of *var* gene transcription profiles was performed using primers and PCR conditions as previously described [86]. In brief, RNA was extracted in Trizol LS (Invitrogen) from ring stage parasites at ∼6–12 hours post-invasion and purified on RNeasy Micro columns with on-column DNaseI treatment (QIAGEN) according to manufacturer's protocols. cDNA was synthesized from 4 µg total RNA using Multi-Scribe reverse transcriptase (Applied Biosystems) and one half of this material was used for each real-time reaction against the complete set of primers. Real-time reactions were performed on an ABI Prism 7500 thermocycler at optimized final primer concentrations of 0.05 µM-0.5 µM using Power-SYBR Green Master Mix in 20 µL reaction volumes under the following PCR conditions: 50°C for 1 min, 95°C for 10 min, then 40 cycles of dissociation, annealing, and extension at 95°C for 15 sec, 52°C for 15 sec, and 60°C for 45 sec, respectively. Relative transcription was determined by normalization to the adenylosuccinate lyase (ASL, PFB0295w) control housekeeping gene. After optimizing primer efficiencies, residual primer bias was corrected by calculating the average difference in C_T_ values between each optimized IT4 *var* primer pair and ASL using genomic DNA as template to provide a final normalized correction.

### Infected erythrocyte binding assays

Parasite RNA was collected and binding assays performed within the same growth cycle to accurately assess *var* transcription at the time of the binding assay. For binding assays, individual CHO cell lines were grown to subconfluent levels on 60-mm tissue culture-treated dishes (BD Falcon) and recombinant proteins were immobilized by overnight incubation onto 60-mm polystyrene dishes (Corning). The following proteins were analyzed: CD36-Fc (R&D Systems), ICAM-1-Fc (R&D Systems), HABP1/gC1qR-6x HIS (R&D Systems), Fractalkine-6x-HIS (R&D Systems), CD31/PECAM-1 (R&D Systems), TSP-1-10x HIS (R&D Systems), and CSA (Sigma). All proteins and CSA were applied at 50 µg/mL except for CD36 and ICAM-1, which were additionally applied at 5 µg/mL and 100 µg/mL. On the day of the assay, dishes containing CHO cells were washed twice with pre-warmed cell binding medium (BM_cell_: RPMI-1640 medium containing 0.1% bovine serum albumin, pH 7.2) and protein spots were blocked with 2% bovine serum albumin for 45 min at 37°C, then washed twice with pre-warmed protein binding medium (BM_protein_: RPMI-1640 medium containing 0.1% bovine serum albumin, pH 6.8). Infected erythrocytes (3-8% parasitemia) were washed and resuspended to 1% hematocrit in either BM_cell_ or BM_protein_ then overlayed onto CHO cells or spotted onto immobilized proteins, respectively, and incubated for 1 hr at 37°C. Prior to binding assays, rosettes in the three UpsA parasite lines were disrupted in binding medium containing 100 Units/mL heparin sulfate (Sigma). The same conditions were used when testing the effect of heparin sulfate on all of the parasites in the panel. In additional assays to test the effect of sulfated glycoconjugates on IE binding, either 10 µg/mL dextran sulfate (MW >500,000; Sigma) or 100 Units/mL heparin sulfate were included during binding assays. Non-binding erythrocytes were removed by gently flooding each dish with warm binding medium, rocking the dish back and forth several times to resuspend non-binding erythrocytes, then pouring off and replacing the medium. The initial washing procedure was performed on CHO745 cells and 2% BSA spots and was repeated until non-binding erythrocytes were sufficiently removed by observation under 400X magnification. The remaining cells and spots then received the same number of washes. For quantification, dishes were fixed in 1% glutaraldehyde for 20 min at room temperature, then stained with 1X Giemsa for 15 minutes. Binding was quantified by determining the number of IE adhering to at least 300 cells under 1000X magnification or the number of IE per mm^2^ in 4 random fields under 400X magnification. All binding assays were repeated in duplicate.

### Flow cytometry analysis

Trophozoite stage infected RBCs were incubated for one hour at room temperature with specific monoclonal mouse antibodies against R29var NTS-DBLα (mAb 3B13C5, 1∶500) Pf13_0003 NTS-DBLα (mAb J3.21, 1∶20), or VarO NTS-DBLα (mAb D15-50, 1∶20). Antibody labeling was detected with goat anti-mouse IgG-R-Phycoerythrin (Sigma) (1∶20) for 30 minutes. Infected erythrocyte nuclei were detected with SYTO 61 DNA dye (Invitrogen) (1∶1000) added with the secondary antibody. Stained cells were washed in PBS and analyzed on an LSRII FACS machine (BD Biosciences). Analysis was performed using FlowJo 8 (Tree Star, Inc).

## Supporting Information

Figure S1 Flow cytometric analysis of infected erythrocytes expressing UpsA PfEMP1 proteins. Infected erythrocytes were labeled with specific monoclonal antibodies made against the NTS-DBLα domain in R29var, Pf13_0003, or VarO PfEMP1 proteins. FACS histograms of gated infected erythrocytes labeled with monoclonal antibodies (blue lines) or without (red lines). The rosetting rate (RR), or the ability of infected erythrocytes to bind non-infected RBCs at the time of antibody labeling, is indicated in parentheses as the percentage of IE forming rosettes with uninfected red blood cells.
(TIF)

Figure S2 Binding to immobilized CD36 in the presence of heparin sulfate. (**A**) Infected erythrocyte binding to triplicate spots of immobilized CD36 protein (50 µg/mL) was compared with or without addition of heparin sulfate (100 U/mL) to the binding medium. (**B**) Rosetting rate for three UpsA parasite variants with and without heparin sulfate (100 U/mL) was determined by live staining of parasite cultures with ethidium bromide (10 µg/mL) followed by fluorescent microscopy. The rosetting rate was calculated as the percentage of fluorescent infected erythrocytes bound to 2 or more non-fluorescent uninfected erythrocytes. (**C**) Comparison of median infected erythrocyte binding to triplicate spots of immobilized CD36 protein (50 µg/mL) with or without addition of heparin sulfate.
(TIF)

Figure S3 CD36 and ICAM-1 binding in the presence of sulfated glycoconjugates. Infected erythrocyte binding was determined for two parasite lines (ItG-ICAM/*ITvar16* and A4ultra/*ITvar14*) without or in the presence of either 100 U/mL heparin sulfate or 10 µg/mL dextran sulfate. (**A**) Infected erythrocyte binding to CHO745 cells and CHO745 cell transfectants expressing either human CD36 or ICAM-1 receptor protein. (**B**) Infected erythrocyte binding to recombinant CD36-Fc or ICAM-1-Fc fusion proteins at 50 µg/mL and to 2% bovine serum albumin employed as a blocking agent. All proteins were immobilized in 10 µL spots onto polystyrene substrate prior to IE binding analysis.
(TIF)

Figure S4 Trypsin-resistant infected erythrocyte binding to recombinant ICAM-1 protein. The *IT4var31*-expressing parasite line P5B6 was either pretreated with 1 mg/mL trypsin or untreated and then tested for binding to immobilized ICAM-1 protein at 50 µg/mL (**A**) or to immobilized CD36 protein at 50 µg/mL (**B**). P5B6-infected erythrocytes displayed trypsin-resistant binding to ICAM-1. Binding could be blocked with a monoclonal antibody to ICAM-1 (mAb 15.2), but not an isotype control antibody.
(TIF)

## References

[1] Kraemer SM, Smith JD (2006). A family affair: var genes, PfEMP1 binding, and malaria
disease.. Curr Opin Micro.

[2] Miller LH, Baruch DI, Marsh K, Doumbo OK (2002). The pathogenic basis of malaria.. Nature.

[3] Frank M, Deitsch K (2006). Activation, silencing and mutually exclusive expression within
the var gene family of Plasmodium falciparum.. Int J Parasitol.

[4] Gardner MJ, Hall N, Fung E, White O, Berriman M (2002). Genome sequence of the human malaria parasite Plasmodium
falciparum.. Nature.

[5] Biggs BA, Anders RF, Dillon HE, Davern KM, Martin M (1992). Adherence of infected erythrocytes to venular endothelium selects
for antigenic variants of Plasmodium falciparum.. J Immunol.

[6] Roberts DJ, Craig AG, Berendt AR, Pinches R, Nash G (1992). Rapid switching to multiple antigenic and adhesive phenotypes in
malaria.. Nature.

[7] Smith JD, Chitnis CE, Craig AG, Roberts DJ, Hudson-Taylor DE (1995). Switches in expression of Plasmodium falciparum var genes
correlate with changes in antigenic and cytoadherent phenotypes of infected
erythrocytes.. Cell.

[8] Freitas-Junior LH, Bottius E, Pirrit LA, Deitsch KW, Scheidig C (2000). Frequent ectopic recombination of virulence factor genes in
telomeric chromosome clusters of P. falciparum.. Nature.

[9] Kraemer SM, Kyes SA, Aggarwal G, Springer AL, Nelson SO (2007). Patterns of gene recombination shape var gene repertoires in
Plasmodium falciparum: comparisons of geographically diverse
isolates.. BMC Genomics.

[10] Rask TS, Hansen DA, Theander TG, Gorm PA, Lavstsen T (2010). Plasmodium falciparum Erythrocyte Membrane Protein 1 Diversity in
Seven Genomes - Divide and Conquer.. PLoS Comput Biol.

[11] Lavstsen T, Salanti A, Jensen AT, Arnot DE, Theander TG (2003). Sub-grouping of Plasmodium falciparum 3D7 var genes based on
sequence analysis of coding and non-coding regions.. Malar J.

[12] Kraemer SM, Smith JD (2003). Evidence for the importance of genetic structuring to the
structural and functional specialization of the Plasmodium falciparum var
gene family.. Mol Microbiol.

[13] Rowe JA, Kyes SA, Rogerson SJ, Babiker HA, Raza A (2002). Identification of a conserved Plasmodium falciparum var gene
implicated in malaria in pregnancy.. J Infect Dis.

[14] Salanti A, Staalsoe T, Lavstsen T, Jensen AT, Sowa MP (2003). Selective upregulation of a single distinctly structured var gene
in chondroitin sulphate A-adhering Plasmodium falciparum involved in
pregnancy-associated malaria.. Mol Microbiol.

[15] Trimnell AR, Kraemer SM, Mukherjee S, Phippard DJ, Janes JH (2006). Global genetic diversity and evolution of var genes associated
with placental and severe childhood malaria.. Mol Biochem Parasitol.

[16] Ho M, Singh B, Looareesuwan S, Davis TM, Bunnag D (1991). Clinical correlates of in vitro Plasmodium falciparum
cytoadherence.. Infect Immun.

[17] Newbold C, Warn P, Black G, Berendt A, Craig A (1997). Receptor-specific adhesion and clinical disease in Plasmodium
falciparum.. Am J Trop Med Hyg.

[18] Ockenhouse CF, Ho M, Tandon NN, Van Seventer GA, Shaw S (1991). Molecular basis of sequestration in severe and uncomplicated
Plasmodium falciparum malaria: differential adhesion of infected
erythrocytes to CD36 and ICAM-1.. J Infect Dis.

[19] Rogerson SJ, Tembenu R, Dobano C, Plitt S, Taylor TE (1999). Cytoadherence characteristics of Plasmodium falciparum-infected
erythrocytes from Malawian children with severe and uncomplicated
malaria.. Am J Trop Med Hyg.

[20] Rowe J, Claessens A, Corrigan R, Arman M (2010). Adhesion of Plasmodium falciparum-infected erythrocytes to human
cells: molecular mechanisms and therapeutic implications.. Expert Rev Mol Med.

[21] Cooke BM, Berendt AR, Craig AG, MacGregor J, Newbold CI (1994). Rolling and stationary cytoadhesion of red blood cells
parasitized by Plasmodium falciparum: separate roles for ICAM-1, CD36 and
thrombospondin.. Br J Haematol.

[22] Gray C, McCormick C, Turner G, Craig A (2003). ICAM-1 can play a major role in mediating P. falciparum adhesion
to endothelium under flow.. Mol Biochem Parasitol.

[23] Ho M, Hickey MJ, Murray AG, Andonegui G, Kubes P (2000). Visualization of Plasmodium falciparum-endothelium interactions
in human microvasculature: mimicry of leukocyte recruitment.. J Exp Med.

[24] Turner GD, Morrison H, Jones M, Davis TM, Looareesuwan S (1994). An immunohistochemical study of the pathology of fatal malaria.
Evidence for widespread endothelial activation and a potential role for
intercellular adhesion molecule-1 in cerebral sequestration.. Am J Pathol.

[25] Fry AE, Auburn S, Diakite M, Green A, Richardson A (2008). Variation in the ICAM1 gene is not associated with severe malaria
phenotypes.. Genes Immun.

[26] Carlson J, Helmby H, Hill AV, Brewster D, Greenwood BM (1990). Human cerebral malaria: association with erythrocyte rosetting
and lack of anti-rosetting antibodies.. Lancet.

[27] Rowe A, Obeiro J, Newbold CI, Marsh K (1995). Plasmodium falciparum rosetting is associated with malaria
severity in Kenya.. Infect Immun.

[28] Treutiger CJ, Hedlund I, Helmby H, Carlson J, Jepson A (1992). Rosette formation in Plasmodium falciparum isolates and
anti-rosette activity of sera from Gambians with cerebral or uncomplicated
malaria.. Am J Trop Med Hyg.

[29] Fried M, Duffy PE (1996). Adherence of Plasmodium falciparum to chondroitin sulfate A in
the human placenta.. Science.

[30] Gupta S, Snow RW, Donnelly CA, Marsh K, Newbold C (1999). Immunity to non-cerebral severe malaria is acquired after one or
two infections.. Nat Med.

[31] Marsh K, Snow RW (1997). Host-parasite interaction and morbidity in malaria endemic
areas.. Philos Trans R Soc Lond B Biol Sci.

[32] Bull PC, Lowe BS, Kortok M, Marsh K (1999). Antibody recognition of Plasmodium falciparum erythrocyte surface
antigens in Kenya: evidence for rare and prevalent variants.. Infect Immun.

[33] Bull PC, Kortok M, Kai O, Ndungu F, Ross A (2000). Plasmodium falciparum-infected erythrocytes: agglutination by
diverse Kenyan plasma is associated with severe disease and young host
age.. J Infect Dis.

[34] Nielsen MA, Staalsoe T, Kurtzhals JA, Goka BQ, Dodoo D (2002). Plasmodium falciparum variant surface antigen expression varies
between isolates causing severe and nonsevere malaria and is modified by
acquired immunity.. J Immunol.

[35] Cham GK, Turner L, Lusingu J, Vestergaard L, Mmbando BP (2009). Sequential, ordered acquisition of antibodies to Plasmodium
falciparum erythrocyte membrane protein 1 domains.. J Immunol.

[36] Cham GK, Turner L, Kurtis JD, Mutabingwa T, Fried M (2010). Hierarchical, domain type-specific acquisition of antibodies to
Plasmodium falciparum erythrocyte membrane protein 1 in Tanzanian
children.. Infect Immun.

[37] Jensen AT, Magistrado P, Sharp S, Joergensen L, Lavstsen T (2004). Plasmodium falciparum associated with severe childhood malaria
preferentially expresses PfEMP1 encoded by group A var
genes.. J Exp Med.

[38] Kyriacou HM, Stone GN, Challis RJ, Raza A, Lyke KE (2006). Differential var gene transcription in Plasmodium falciparum
isolates from patients with cerebral malaria compared to
hyperparasitaemia.. Mol Biochem Parasitol.

[39] Warimwe GM, Keane TM, Fegan G, Musyoki JN, Newton CR (2009). Plasmodium falciparum var gene expression is modified by host
immunity.. Proc Natl Acad Sci U S A.

[40] Smith JD, Subramanian G, Gamain B, Baruch DI, Miller LH (2000). Classification of adhesive domains in the Plasmodium falciparum
erythrocyte membrane protein 1 family.. Mol Biochem Parasitol.

[41] Baruch DI, Pasloske BL, Singh HB, Bi X, Ma XC (1995). Cloning the P. falciparum gene encoding PfEMP1, a malarial
variant antigen and adherence receptor on the surface of parasitized human
erythrocytes.. Cell.

[42] Su XZ, Heatwole VM, Wertheimer SP, Guinet F, Herrfeldt JA (1995). The large diverse gene family var encodes proteins involved in
cytoadherence and antigenic variation of Plasmodium falciparum-infected
erythrocytes.. Cell.

[43] Smith JD, Gamain B, Baruch DI, Kyes S (2001). Decoding the language of var genes and Plasmodium falciparum
sequestration.. Trends Parasitol.

[44] Howell DP, Levin EA, Springer AL, Kraemer SM, Phippard DJ (2008). Mapping a common interaction site used by Plasmodium falciparum
Duffy binding-like domains to bind diverse host receptors.. Mol Microbiol.

[45] Oleinikov AV, Amos E, Frye IT, Rossnagle E, Mutabingwa TK (2009). High throughput functional assays of the variant antigen PfEMP1
reveal a single domain in the 3D7 Plasmodium falciparum genome that binds
ICAM1 with high affinity and is targeted by naturally acquired neutralizing
antibodies.. PLoS Pathog.

[46] Robinson BA, Welch TL, Smith JD (2003). Widespread functional specialization of Plasmodium falciparum
erythrocyte membrane protein 1 family members to bind CD36 analysed across a
parasite genome.. Mol Microbiol.

[47] Joergensen L, Bengtsson DC, Bengtsson A, Ronander E, Berger SS (2010). Surface co-expression of two different PfEMP1 antigens on single
Plasmodium falciparum-infected erythrocytes facilitates binding to ICAM1 and
PECAM1.. PLoS Pathog.

[48] Gamain B, Gratepanche S, Miller LH, Baruch DI (2002). Molecular basis for the dichotomy in Plasmodium falciparum
adhesion to CD36 and chondroitin sulfate A.. Proc Natl Acad Sci U S A.

[49] Dahlback M, Nielsen MA, Salanti A (2010). Can any lessons be learned from the ambiguous glycan binding of
PfEMP1 domains?. Trends Parasitol.

[50] Bourke PF, Holt DC, Sutherland CJ, Kemp DJ (1996). Disruption of a novel open reading frame of Plasmodium falciparum
chromosome 9 by subtelomeric and internal deletions can lead to loss or
maintenance of cytoadherence.. Mol Biochem Parasitol.

[51] Udeinya IJ, Graves PM, Carter R, Aikawa M, Miller LH (1983). Plasmodium falciparum: effect of time in continuous culture on
binding to human endothelial cells and amelanotic melanoma
cells.. Exp Parasitol.

[52] Horrocks P, Pinches R, Christodoulou Z, Kyes SA, Newbold CI (2004). Variable var transition rates underlie antigenic variation in
malaria.. Proc Natl Acad Sci U S A.

[53] Vigan-Womas I, Guillotte M, Juillerat A, Vallieres C, Lewit-Bentley A (2011). Allelic diversity of the Plasmodium falciparum erythrocyte
membrane protein 1 entails variant-specific red cell surface
epitopes.. PLoS One.

[54] Crabb BS, Cooke BM, Reeder JC, Waller RF, Caruana SR (1997). Targeted gene disruption shows that knobs enable malaria-infected
red cells to cytoadhere under physiological shear stress.. Cell.

[55] Rug M, Prescott SW, Fernandez KM, Cooke BM, Cowman AF (2006). The role of KAHRP domains in knob formation and cytoadherence of
P falciparum-infected human erythrocytes.. Blood.

[56] McCormick CJ, Newbold CI, Berendt AR (2000). Sulfated glycoconjugates enhance CD36-dependent adhesion of
Plasmodium falciparum-infected erythrocytes to human microvascular
endothelial cells.. Blood.

[57] Gardner JP, Pinches RA, Roberts DJ, Newbold CI (1996). Variant antigens and endothelial receptor adhesion in Plasmodium
falciparum.. Proc Natl Acad Sci U S A.

[58] Smith JD, Craig AG, Kriek N, Hudson-Taylor D, Kyes S (2000). Identification of a Plasmodium falciparum intercellular adhesion
molecule-1 binding domain: a parasite adhesion trait implicated in cerebral
malaria.. Proc Natl Acad Sci U S A.

[59] Chattopadhyay R, Taneja T, Chakrabarti K, Pillai CR, Chitnis CE (2004). Molecular analysis of the cytoadherence phenotype of a Plasmodium
falciparum field isolate that binds intercellular adhesion
molecule-1.. Mol Biochem Parasitol.

[60] Duffy MF, Maier AG, Byrne TJ, Marty AJ, Elliott SR (2006). VAR2CSA is the principal ligand for chondroitin sulfate A in two
allogeneic isolates of Plasmodium falciparum.. Mol Biochem Parasitol.

[61] Viebig NK, Gamain B, Scheidig C, Lepolard C, Przyborski J (2005). A single member of the Plasmodium falciparum var multigene family
determines cytoadhesion to the placental receptor chondroitin sulphate
A.. EMBO Rep.

[62] Smith JD, Deitsch KW (2004). Pregnancy-associated malaria and the prospects for
syndrome-specific antimalaria vaccines.. J Exp Med.

[63] Magistrado P, Salanti A, Tuikue Ndam NG, Mwakalinga SB, Resende M (2008). VAR2CSA expression on the surface of placenta-derived Plasmodium
falciparum-infected erythrocytes.. J Infect Dis.

[64] Peters JM, Fowler EV, Krause DR, Cheng Q, Gatton ML (2007). Differential Changes in Plasmodium falciparum var Transcription
during Adaptation to Culture.. The J Infect Dis.

[65] Frank M, Dzikowski R, Amulic B, Deitsch K (2007). Variable switching rates of malaria virulence genes are
associated with chromosomal position.. Mol Microbiol.

[66] Baruch DI, Ma XC, Singh HB, Bi X, Pasloske BL (1997). Identification of a region of PfEMP1 that mediates adherence of
Plasmodium falciparum infected erythrocytes to CD36: conserved function with
variant sequence.. Blood.

[67] Crandall I, Land KM, Sherman IW (1994). Plasmodium falciparum: pfalhesin and CD36 form an
adhesin/receptor pair that is responsible for the pH-dependent portion of
cytoadherence/sequestration.. Exp Parasitol.

[68] Ockenhouse CF, Klotz FW, Tandon NN, Jamieson GA (1991). Sequestrin, a CD36 recognition protein on Plasmodium falciparum
malaria- infected erythrocytes identified by anti-idiotype
antibodies.. Proc Natl Acad Sci U S A.

[69] Handunnetti SM, van Schravendijk MR, Hasler T, Barnwell JW, Greenwalt DE, Howard RJ (1992). Involvement of CD36 on erythrocytes as a rosetting receptor for
Plasmodium falciparum-infected erythrocytes.. Blood.

[70] Magistrado PA, Staalsoe T, Theander TG, Hviid L, Jensen AT (2008). CD36 selection of 3D7 Plasmodium falciparum associated with
severe childhood malaria results in reduced VAR4 expression.. Malar J.

[71] H, Behr C, Mercereau-Puijalon O, Michel J (2000). Plasmodium falciparum in the squirrel monkey (Saimiri sciureus):
infection of non-splenectomised animals as a model for exploring clinical
manifestations of malaria.. Microbes Infect.

[72] Kaul DK, Roth EF, Nagel RL, Howard RJ, Handunnetti SM (1991). Rosetting of Plasmodium falciparum-infected red blood cells with
uninfected red blood cells enhances microvascular obstruction under flow
conditions.. Blood.

[73] Springer AL, Smith LM, Mackay DQ, Nelson SO, Smith JD (2004). Functional interdependence of the DBLbeta domain and c2 region
for binding of the Plasmodium falciparum variant antigen to
ICAM-1.. Mol Biochem Parasitol.

[74] Urban BC, Ferguson DJ, Pain A, Willcox N, Plebanski M (1999). Plasmodium falciparum-infected erythrocytes modulate the
maturation of dendritic cells.. Nature.

[75] Urban BC, Roberts DJ (2002). Malaria, monocytes, macrophages and myeloid dendritic cells:
sticking of infected erythrocytes switches off host cells.. Curr Opin Immunol.

[76] Melcher M, Muhle RA, Henrich PP, Kraemer SM, Avril M (2010). Identification of a role for the PfEMP1 semi-conserved head
structure in protein trafficking to the surface of Plasmodium falciparum
infected red blood cells.. Cell Microbiol.

[77] Patel SN, Lu Z, Ayi K, Serghides L, Gowda DC (2007). Disruption of CD36 impairs cytokine response to Plasmodium
falciparum glycosylphosphatidylinositol and confers susceptibility to severe
and fatal malaria in vivo.. J Immunol.

[78] Serghides L, Smith TG, Patel SN, Kain KC (2003). CD36 and malaria: friends or foes?. Trends Parasitol.

[79] Kalmbach Y, Rottmann M, Kombila M, Kremsner PG, Beck HP (2010). Differential var gene expression in children with malaria and
antidromic effects on host gene expression.. J Infect Dis.

[80] Kirchgatter K, Portillo Hdel A (2002). Association of severe noncerebral Plasmodium falciparum malaria
in Brazil with expressed PfEMP1 DBL1 alpha sequences lacking cysteine
residues.. Mol Med.

[81] Rottmann M, Lavstsen T, Mugasa JP, Kaestli M, Jensen ATR (2006). Differential Expression of var Gene Groups Is Associated with
Morbidity Caused by Plasmodium falciparum Infection in Tanzanian
Children.. Infect Immun.

[82] Vigan-Womas I, Guillotte M, Le SC, Igonet S, Petres S (2008). An in vivo and in vitro model of Plasmodium falciparum rosetting
and autoagglutination mediated by varO, a group A var gene encoding a
frequent serotype.. Infect Immun.

[83] Rowe JA, Moulds JM, Newbold CI, Miller LH (1997). P. falciparum rosetting mediated by a parasite-variant
erythrocyte membrane protein and complement-receptor 1.. Nature.

[84] Reeder JC, Cowman AF, Davern KM, Beeson JG, Thompson JK (1999). The adhesion of Plasmodium falciparum-infected erythrocytes to
chondroitin sulfate A is mediated by P. falciparum erythrocyte membrane
protein 1.. Proc Natl Acad Sci U S A.

[85] Buffet PA, Gamain B, Scheidig C, Baruch D, Smith JD (1999). Plasmodium falciparum domain mediating adhesion to chondroitin
sulfate A: a receptor for human placental infection.. Proc Natl Acad Sci U S A.

[86] Viebig NK, Levin E, Dechavanne S, Rogerson SJ, Gysin J (2007). Disruption of var2csa gene impairs placental malaria associated
adhesion phenotype.. PLoS One.

