# Supplementary figures and images for: Investigating the Host Binding Signature on the Plasmodium falciparum PfEMP1 Protein Family
Source: PLoS Pathog. 2011 May 5;7(5):e1002032. doi: 10.1371/journal.ppat.1002032 (PMC3088720; doi:10.1371/journal.ppat.1002032)

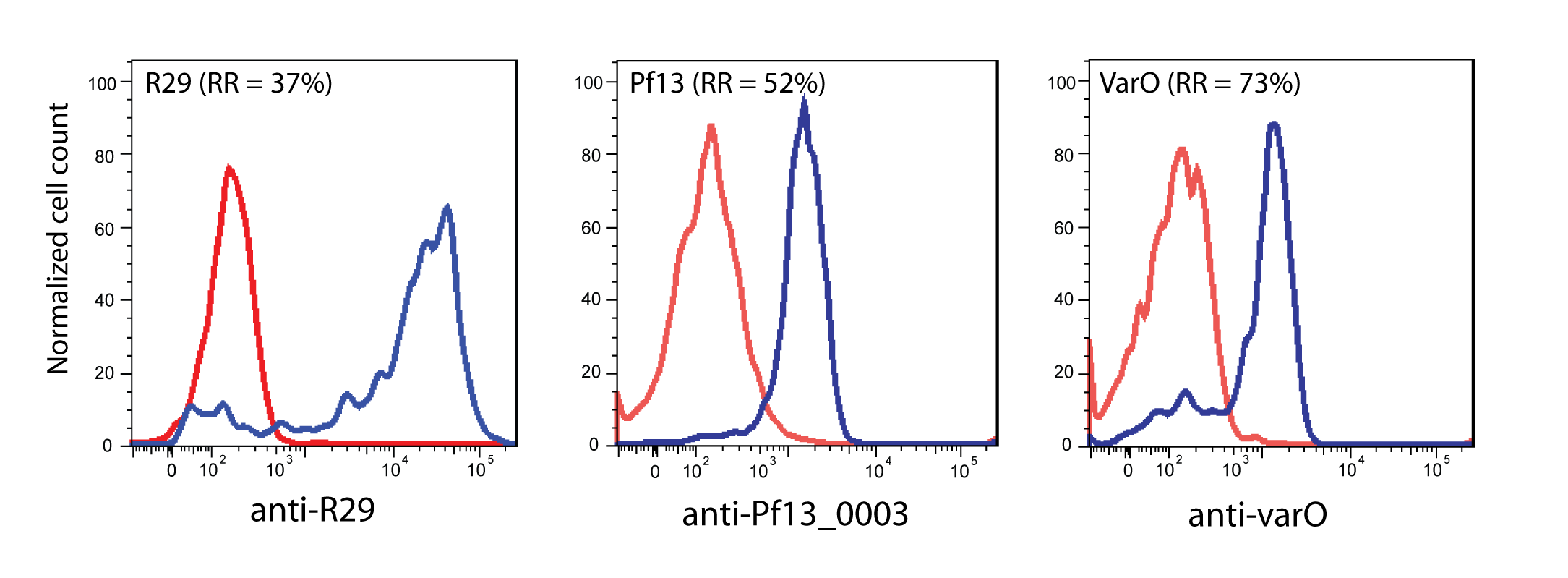

Supplement: Figure S1 — Flow cytometric analysis of infected erythrocytes expressing UpsA PfEMP1 proteins. Infected erythrocytes were labeled with specific monoclonal antibodies made against the NTS-DBLα domain in R29var, Pf13_0003, or VarO PfEMP1 proteins. FACS histograms of gated infected erythrocytes labeled with monoclonal antibodies (blue lines) or without (red lines). The rosetting rate (RR), or the ability of infected erythrocytes to bind non-infected RBCs at the time of antibody labeling, is indicated in parentheses as the percentage of IE forming rosettes with uninfected red blood cells. (TIF) [file ppat.1002032.s001.tif]

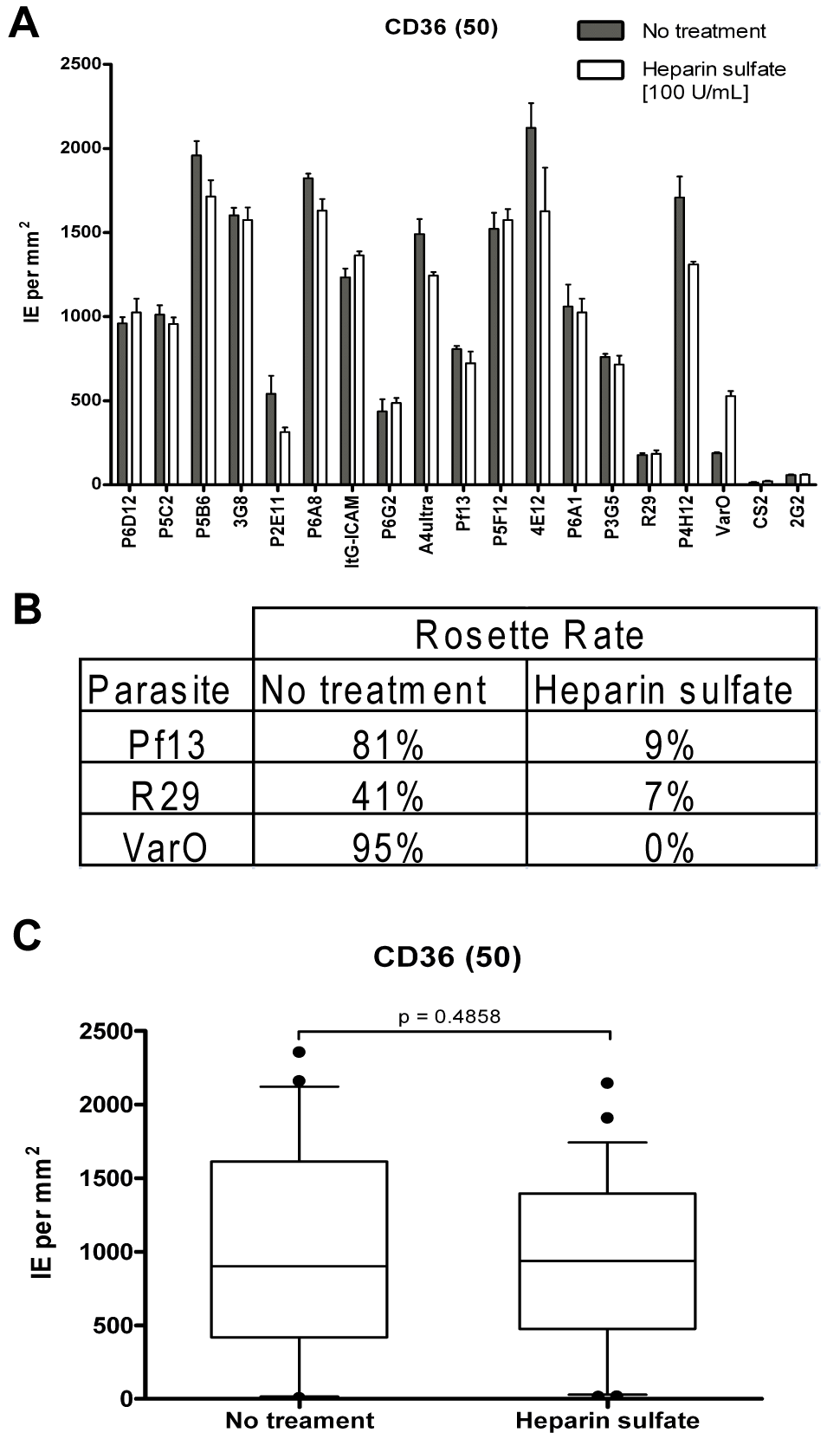

Supplement: Figure S2 — Binding to immobilized CD36 in the presence of heparin sulfate. (A) Infected erythrocyte binding to triplicate spots of immobilized CD36 protein (50 µg/mL) was compared with or without addition of heparin sulfate (100 U/mL) to the binding medium. (B) Rosetting rate for three UpsA parasite variants with and without heparin sulfate (100 U/mL) was determined by live staining of parasite cultures with ethidium bromide (10 µg/mL) followed by fluorescent microscopy. The rosetting rate was calculated as the percentage of fluorescent infected erythrocytes bound to 2 or more non-fluorescent uninfected erythrocytes. (C) Comparison of median infected erythrocyte binding to triplicate spots of immobilized CD36 protein (50 µg/mL) with or without addition of heparin sulfate. (TIF) [file ppat.1002032.s002.tif]

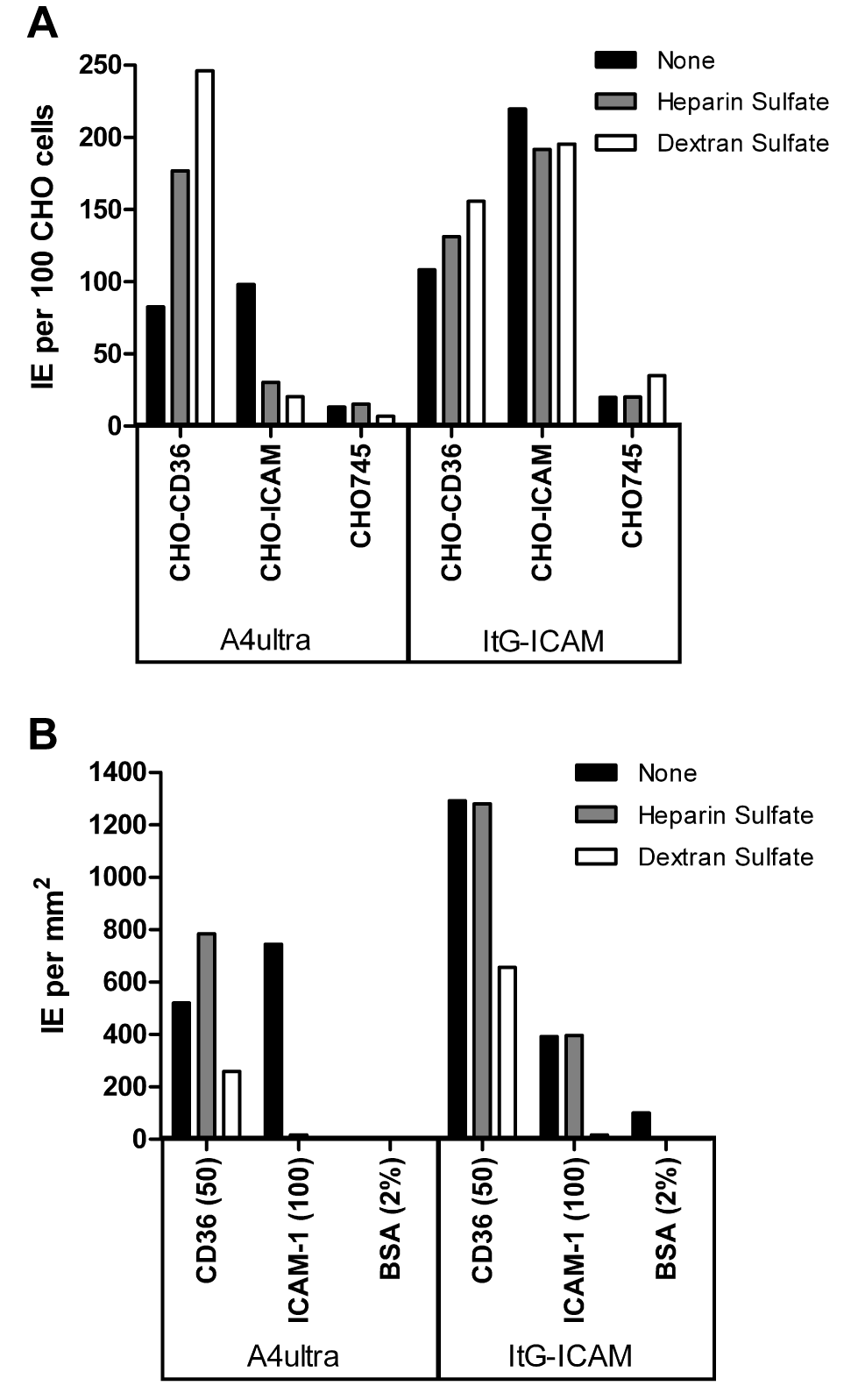

Supplement: Figure S3 — CD36 and ICAM-1 binding in the presence of sulfated glycoconjugates. Infected erythrocyte binding was determined for two parasite lines (ItG-ICAM/ITvar16 and A4ultra/ITvar14) without or in the presence of either 100 U/mL heparin sulfate or 10 µg/mL dextran sulfate. (A) Infected erythrocyte binding to CHO745 cells and CHO745 cell transfectants expressing either human CD36 or ICAM-1 receptor protein. (B) Infected erythrocyte binding to recombinant CD36-Fc or ICAM-1-Fc fusion proteins at 50 µg/mL and to 2% bovine serum albumin employed as a blocking agent. All proteins were immobilized in 10 µL spots onto polystyrene substrate prior to IE binding analysis. (TIF) [file ppat.1002032.s003.tif]

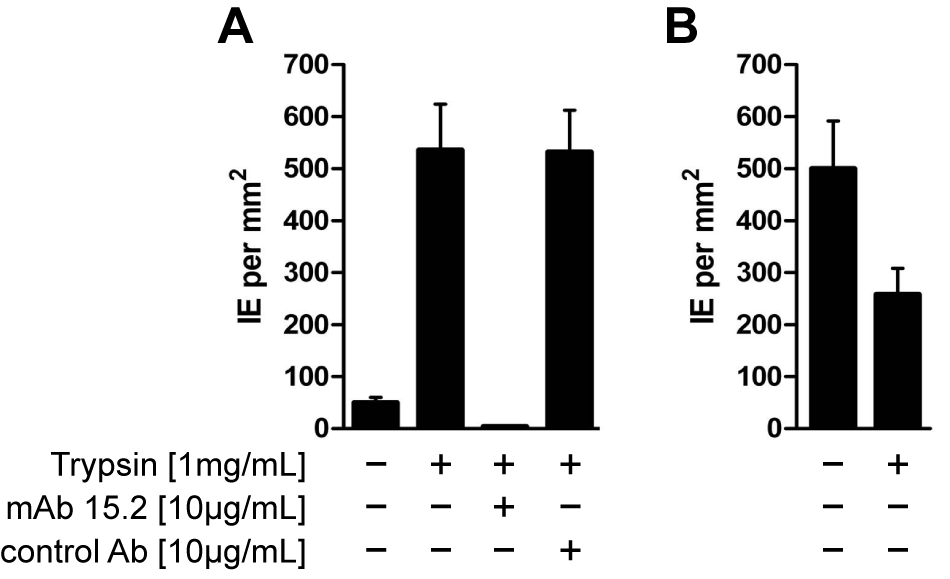

Supplement: Figure S4 — Trypsin-resistant infected erythrocyte binding to recombinant ICAM-1 protein. The IT4var31-expressing parasite line P5B6 was either pretreated with 1 mg/mL trypsin or untreated and then tested for binding to immobilized ICAM-1 protein at 50 µg/mL (A) or to immobilized CD36 protein at 50 µg/mL (B). P5B6-infected erythrocytes displayed trypsin-resistant binding to ICAM-1. Binding could be blocked with a monoclonal antibody to ICAM-1 (mAb 15.2), but not an isotype control antibody. (TIF) [file ppat.1002032.s004.tif]
